# Metabolic pathways of Alternative Lengthening of Telomeres in pan-carcinoma

**DOI:** 10.1371/journal.pone.0314012

**Published:** 2025-02-21

**Authors:** Isaac Armendáriz-Castillo, Jennyfer García-Cárdenas, Pamela Espinosa, Katherine Hidalgo-Fernández, Lizbeth Peña-Zúñiga, Ronie Martínez, Juan Moromenacho, Andrés Herrera-Yela, Jonathan Cruz-Varela, Anilú Saucedo-Sariñana, María-Esperanza Cerdán, Andrés López-Cortés, Santiago Guerrero

**Affiliations:** 1 Laboratorio de Ciencia de Datos Biomédicos, Facultad de Ciencias Médicas de la Salud y de la Vida, Universidad Internacional del Ecuador, Quito, Ecuador; 2 Centro Interdisciplinar de Química e Bioloxía (CICA), Campus de Elviña, Universidade da Coruña, A Coruña, Spain; 3 Instituto Nacional de Investigación en Salud Pública, Quito, Ecuador; 4 Facultad de Ciencias Técnicas, Universidad Internacional del Ecuador, Quito, Ecuador; 5 Experimental and Applied Biomedicine Research Group, Health Sciences Faculty, Universidad Internacional SEK (UISEK), Quito, Ecuador; 6 Group of Emerging and Neglected Diseases, Ecoepidemiology and Biodiversity, Health Sciences Faculty, Universidad Internacional SEK, Quito, Ecuador; 7 School of Biological Sciences & Engineering, Universidad Yachay Tech, Urcuqui, Ecuador; 8 Departamento Académico de Aparatos y Sistemas I, Universidad Autónoma de Guadalajara, Zapopan, México; 9 Cancer Research Group (CRG), Faculty of Medicine, Universidad de Las Américas, Quito, Ecuador; Tulane University Health Sciences Center, UNITED STATES OF AMERICA

## Abstract

Alternative Lengthening of Telomeres (ALT) is a telomerase-independent mechanism deployed by several aggressive cancers to maintain telomere length. This contributes to their malignancy and resistance to conventional therapies. In prior studies, we have identified key proteins linked to the ALT process using multi-omic data integration strategies. In this work, we combined metabolomic datasets with our earlier results to identify targetable metabolic pathways for ALT-positive tumors. 39 ALT-related proteins were found to interact with 42 different metabolites in our analysis. Additional networking analysis revealed a complex interaction between metabolites and ALT-related proteins, suggesting that pan-cancer oncogenes may have an impact on these pathways. Three metabolic pathways have been primarily related with the ALT mechanism: purine metabolism, cysteine and methionine metabolism, and nicotinate and nicotinamide metabolism. Lastly, we prioritized FDA-approved drugs (azathioprine, thioguanine, and mercaptopurine) that could target ALT-positive tumors through purine metabolism. This work provides a wide perspective of the metabolomic pathways associated with ALT and reveals potential therapeutic targets that require further experimental validation.

## Introduction

Telomeres are nucleoprotein complexes that, in tandem with shelterin complex, protect the ends of eukaryotic chromosomes from degradation, recombination, and end to end fusion [1]. Each round of the cellular cycle results in the shortening of telomeres, a process that, in healthy somatic cells, functions as a natural mechanism of cellular senescence [2, 3]. In cancer, most tumors activate various telomere maintenance mechanisms (TMM) that enable them to elongate their telomeres and achieve cellular immortality [4]. Thus, a variety of therapies aimed at blocking these mechanisms have been implemented in recent years [2, 4].

Despite these efforts, approximately 15% of cancers can activate another TMM known as the Alternative Lengthening of Telomeres (ALT) [5]. ALT is a telomerase-independent mechanism that utilizes homologous recombination to extend telomeres [5]. While ALT is commonly associated with tumors of mesenchymal origin, such as sarcomas [6–8], there have been reports of its presence in other tumor types, including carcinomas. ALT+ cells have been identified in carcinomas of breast, liver, lung, bladder, cervix, endometrium, esophagus, and kidney [9–11]. Additionally, ALT activity has been observed in cell lines derived from patients with breast and colon carcinomas, as well in histological analyses of both pediatric and adult cancers, including neuroblastomas and astrocytomas [11]. In fact, a significant proportion of astrocytomas (34%) exhibit ALT activity [12, 13].

Moreover, *in vivo* and *in vitro* experiments have demonstrated the coexistence of ALT with telomerase-dependent mechanisms within the same population of cancer cells [13–15]. It is believed that some cancer cells may switch from a telomerase-dependent mechanism to ALT under selective pressure from telomerase inhibitors [14, 16]. This phenomenon might add an extra layer of complexity to the diagnosis, prognosis, and treatment of ALT-positive cancer patients [16, 17].

To shed light on the ALT molecular mechanism, Braun et al. (2018) have developed the TelNet database [18], which compiles information on over 2000 human genes involved in telomere maintenance, including those specifically associated with the ALT pathway. Since the ALT pathway involves ~400 genes, occurs in multiple cancer types, and is linked to clinical outcomes, we previously prioritized ALT-related genes based on their alterations across 31 cancer types. Thus, we were able to identify 20 genes with significant alterations and proposed these as biomarkers for the ALT process. Additionally, we integrated multi-omic data to investigate 71 APBs (ALT-associated PML bodies)-related genes, uncovering 13 key proteins with distinctive mutations, interactions, and functional patterns related to ALT [19, 20].

To further incorporate metabolomics data into our prior findings, here we perform an integrative analysis of ALT-related proteins and metabolites to reveal putative metabolic pathways implicated in the ALT process, with the potential to be used as biomarkers or therapeutic targets for ALT-positive tumors.

## Methods

### ALT and CDG gene set

ALT related proteins were extracted from TelNet database [18], (http://www.cancertelsys.org/telnet/): 411 were directly associated with the ALT process, and 71 were linked with the ALT-associated PML bodies (S1 Fig). Cancer driver genes (CDGs) [21] were filtered by type (*Pan-cancer*), including *pan-cancer_adult* and *pan-cancer_paediatric* subdivisions and by *NCG_oncogene*. A total of 106 CDGs were filtered.

### Prioritization of ALT-related proteins

A total of 482 ALT-related proteins were prioritized based on their degree of alteration in both pan-cancer and mesenchymal-origin tumors. For pan-cancer analysis, these proteins were evaluated for genomic, transcriptomic, and proteomic alterations across 32 studies from the TCGA Pan Cancer Atlas (n = 10,918 samples), as previously reported [19, 20]. For mesenchymal-origin tumors, genomic alterations (mutations, structural variants, and copy number alterations) were extracted from 17 studies encompassing 12,405 samples (S1 Table in S1 File) using the cBioPortal platform (https://www.cbioportal.org/). Transcriptomic and proteomic data were unavailable in these datasets. Proteins were ranked according to their number of alterations, and the top quartile (Q1) was selected for further analysis (S1 Fig).

### Detection of ALT-related metabolites

To determine the metabolites implicated in ALT process, we indirectly identified which metabolites are interacting with our previously prioritized ALT-related proteins. Thus, we interrogated the Human Metabolome Database (https://hmdb.ca/downloads) [22]. The interactions of these proteins with their corresponding metabolites were subsequently extracted from the “All Proteins” file using xml2 package (Wickham H, Hester J, Ooms J (2023). xml2: Parse XML. https://xml2.r-lib.org/, https://github.com/r-lib/xml2.) in RStudio 2023.06.1+524 "Mountain Hydrangea" Release (547dcf861cac0253a8abb52c135e44e02ba407a1, 2023-07-07) for windows. Mozilla/5.0 (Windows NT 10.0; Win64; x64) AppleWebKit/537.36 (KHTML, like Gecko) RStudio/2023.06.1+524. Chrome/110.0.5481.208 Electron/23.3.0 Safari/537.36. “All Proteins” file, released on 2021-11-09 (Size: 34.7 MB) was downloaded on 11/10/2023.

### Enrichment analysis of ALT-related metabolites

To detect enriched metabolic pathways associated with the natural metabolites previously prioritized (n = 30), an enrichment analysis was performed using MetaboAnalyst 5.0 [23]. The Small Molecule Pathway Database (SMPDB) [24] and the Kyoto Encyclopedia of Genes and Genomes (KEGG) PATHWAY Database [25] were used for this analysis. To detect druggable metabolic pathways against ALT-positive tumors, we also performed an enrichment analysis in MetaboAnalyst 5.0 [23] of the 30 naturally occurring ALT-related metabolites but using 461 metabolite sets based on drug pathways from SMPDB [24].

### Joint pathway analysis

This integrative analysis was performed in the MetaboAnalyst 5.0 platform [23] using two datasets: 1) 39 ALT-related genes, and 2) 30 naturally occurring metabolites associated with the ALT process. The KEGG PATHWAY Database (Kyoto Encyclopedia of Genes and Genomes) [25] was used for this analysis. Parameters: 1) metabolomics type = targeted (compound list); 2) All pathways (integrated); 3) enrichment analysis = hypergeometric Test; 4) topology measure = degree centrality; 5) integration method = combine queries [23, 26].

### Protein-metabolite interaction network

Cytoscape v3.9.1 [27] was used to generate an interaction network of the proteins and their associated metabolites. This network was constructed based protein-metabolite and protein-protein interactions (PMIs, PPIs) between ALT-related proteins, pan-cancer oncogenes, and their corresponding metabolites. PPIs were obtained from STRING database [28]. The interactions were sourced from both experiments and databases with an interaction score of 0.9. This is the highest possible confidence of an interaction to be true based on all the available evidence. PMIs were extracted from the “All Proteins” file using xml2 package (Wickham H, Hester J, Ooms J (2023). xml2: Parse XML. https://xml2.r-lib.org/, https://github.com/r-lib/xml2.) in RStudio 2023.06.1+524 "Mountain Hydrangea" Release (547dcf861cac0253a8abb52c135e44e02ba407a1, 2023-07-07) for windows. Mozilla/5.0 (Windows NT 10.0; Win64; x64) AppleWebKit/537.36 (KHTML, like Gecko) RStudio/2023.06.1+524. Chrome/110.0.5481.208 Electron/23.3.0 Safari/537.36.

## Results

### Identifying ALT-related metabolic pathways

#### ALT-related metabolites

To elucidate the metabolites implicated in the ALT process and further identify key metabolic pathways in pan-cancer, an indirect analysis was conducted. This analysis aimed to determine which specific metabolites engage in interactions with the ALT-related proteins that had been prioritized in previous stages of our research [19, 20]. These proteins were prioritized by multi-omic *in silico* analyses from our previous studies across 32 cancer types [19, 20] (S1 Fig).

To identify the metabolites linked with these proteins, the Human Metabolome Database was downloaded and interrogated [22]. Thus, we identified a total of 39 ALT-related proteins interacting with 42 distinct metabolites (30 natural and 12 unnatural metabolites) (S2 and S3 Tables in S1 File). Deregulation of some of these metabolites has been correlated to cancer, specifically to colorectal cancer, bladder cancer, and oral squamous cell carcinoma (S2 Table in S1 File).

#### ALT metabolic pathways

To detect specific metabolic pathways related with these natural metabolites (n = 30), we next performed an enrichment analysis using MetaboAnalyst 5.0 [23] with two databases, KEGG [25] and SMPDB [24]. The platform did not find pathway records for three metabolites: HMDB0251846, HMDB0059656 and HMDB0256910.

**Fig 1:**
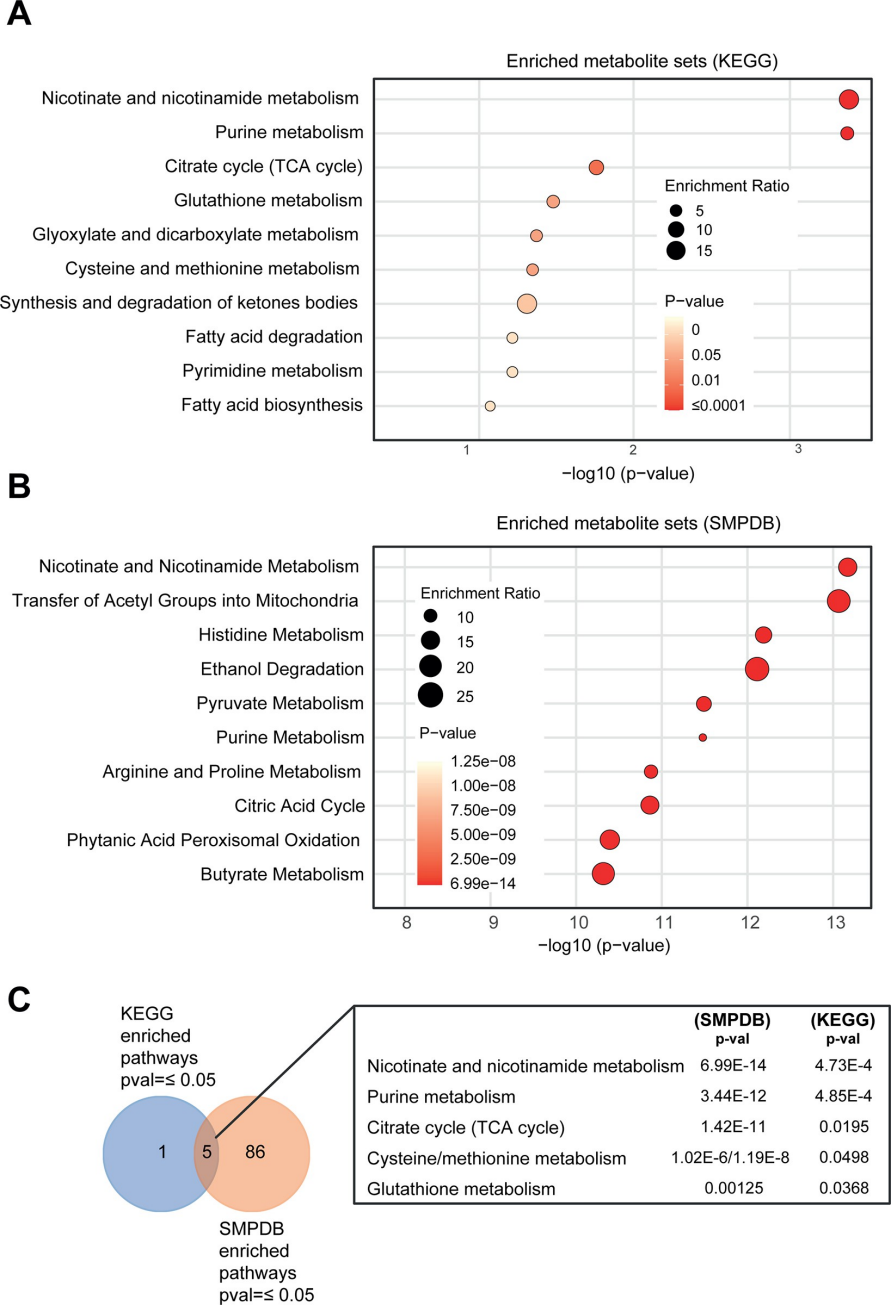
ALT-related metabolic pathways in pan-cancer. Enrichment analysis of ALT-related metabolites using KEGG (A) and SMPDB (B) databases. Metabolite pathways are listed on the y-axis with their respective significance levels shown on the x-axis, represented as the negative logarithm (base 10) of the p-value. The size of the circles is proportional to the enrichment ratio, with larger circles indicating higher enrichment. (C) Venn diagram displaying shared statistically significant pathways between SMPDB and KEGG.

Regarding the KEGG database, we have identified 6 statistically significant metabolic pathways: 1) nicotinate and nicotinamide metabolism (p-val ≤ 0.001), 2) purine metabolism (p-val ≤ 0.001), 3) citrate cycle (TCA cycle) (p-val ≤ 0.05), 4) glutathione metabolism (p-val ≤ 0.05), 5) glyoxylate and dicarboxylate metabolism (p-val ≤ 0.05), and 6) cysteine and methionine metabolism (p-val ≤ 0.05) (Fig 1A; S4 Table in S1 File). Concerning the analysis of the SMPDB, we have identified 92 statistically significant metabolic pathways, 5 with p-values ≤ 0.05, 8 with p-values ≤ 0.01, and 79 with p-values ≤ 0.001 (Fig 1B; S4 Table in S1 File). We finally merged both statistically significant pathway lists from SMPDB and KEGG databases and obtained a final list of 5 metabolic pathways (Fig 1C; S4 Table in S1 File). Table 1 shows the enriched metabolites associated with these pathways.

**Table 1:** Enriched metabolites by pathway and database type.

Metabolic pathway	KEGG	SMPDB
Nicotinate and nicotinamide metabolism	NAD, Niacinamide, and NADP.	Adenosine monophosphate, Pyrophosphate, Adenosine triphosphate, NADP, Magnesium, NAD, S-Adenosylhomocysteine, S-Adenosylmethionine, ADP, Niacinamide, Phosphate, NADH, and Water.
Purine Metabolism	Adenosine triphosphate, ADP, Adenosine monophosphate, dGTP, and Deoxyadenosine triphosphate.	Adenosine monophosphate, Cyclic AMP, NADP, Pyrophosphate, Calcium, Adenosine triphosphate, Magnesium, NAD, Phosphate, dGTP, NADH, Deoxyadenosine triphosphate, Water, and Heme
Citrate cycle (TCA cycle)	Acetyl-CoA, and Citric acid.	Citric acid, Adenosine triphosphate, Magnesium, NAD, Acetyl-CoA, Manganese, ADP, Coenzyme A, Phosphate, NADH, and Water.
Cysteine and methionine metabolism	S-Adenosylmethionine, S-Adenosylhomocysteine.	Adenosine monophosphate, Pyrophosphate, Adenosine triphosphate, Magnesium, NAD, S-Adenosylhomocysteine, S-Adenosylmethionine, Phosphate, NADH, and Water.
Glutathione metabolism	NADP, and Acetyl-CoA.	NADP, Adenosine triphosphate, ADP, and Water.

Given that the ALT process has been observed in tumors of mesenchymal origin, we also prioritized the 482 ALT genes based on their genomic alterations, specifically within a cohort of 12,405 mesenchymal tissue tumor samples (S1 Table in S1 File). We then selected the top quartile (Q1) based on their genomic alterations (S5 Table in S1 File) to indirectly identify metabolic pathways associated with ALT in this tumor type (S6 and S7 Tables in S1 File). Although transcriptomic and proteomic data were not available, we observed similar results (S2 Fig). Thus, by integrating statistically significant pathways from the SMPDB and KEGG databases, we identified five key metabolic pathways: purine metabolism, citrate cycle (TCA cycle), pyruvate metabolism, glycolysis/gluconeogenesis, and nicotinate and nicotinamide metabolism. (S2C Fig).

#### Joint-pathway analysis of ALT-related proteins and metabolites

**Fig 2:**
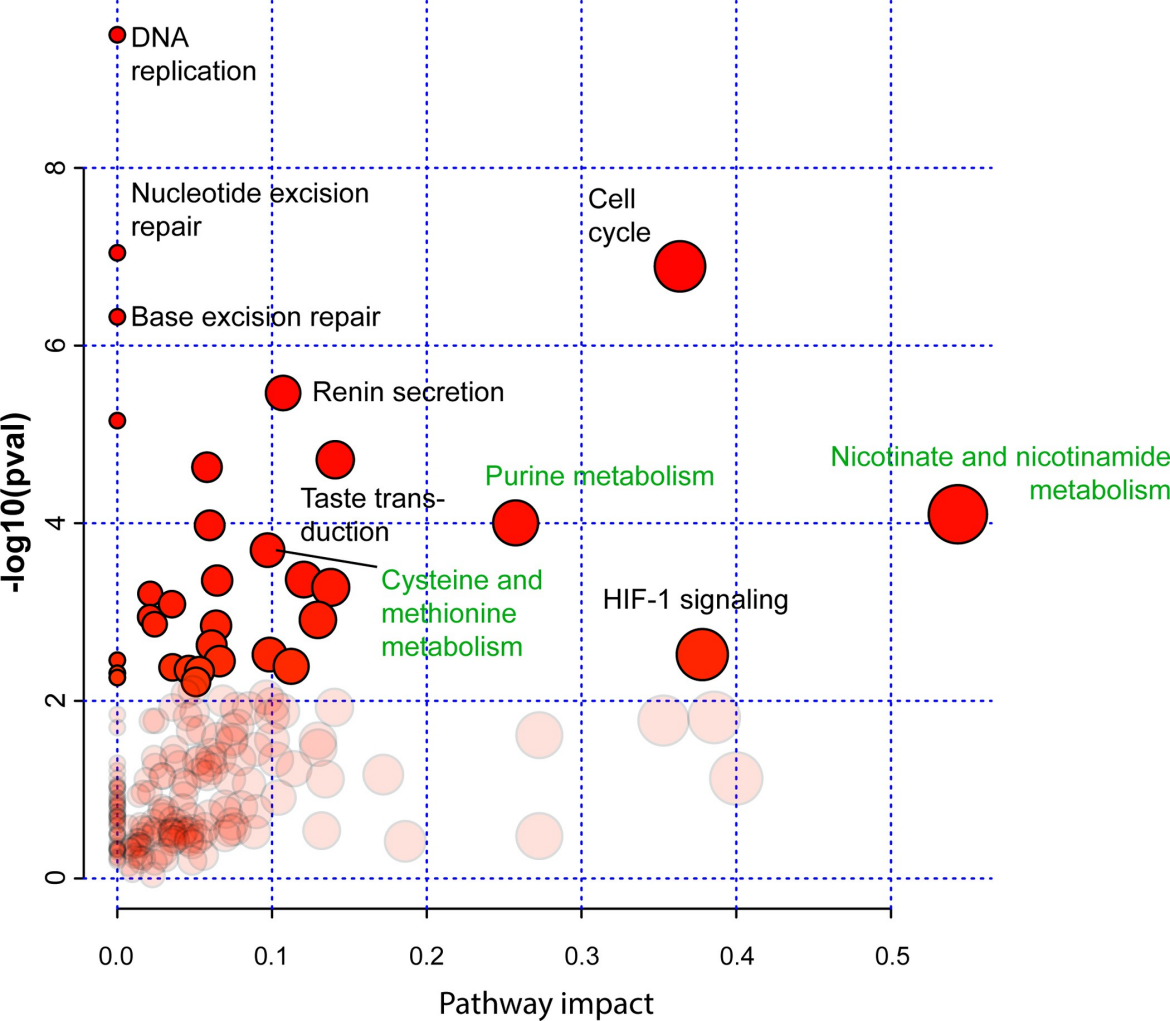
Joint-pathway analysis of ALT-related proteins and metabolites. Displayed is a scatter plot that highlights the metabolic and cellular pathways enriched by the combined presence of ALT-associated proteins and metabolites. The *y* axis shows −log_10_(*P*) values of the enrichment analysis results using the MetaboAnalyst 5.0 platform [23]. The x-axis displays pathway impact values [26], which represent the adjusted topology metrics of the disrupted genes/metabolites within each pathway. The size of the circles is proportional to the enrichment ratio, with larger circles indicating higher enrichment.

To detect enriched metabolic and cellular pathways based on the presence of both genes and metabolites, we performed a joint pathway analysis using MetaboAnalyst 5.0 [23]. We interrogated the database with the 39 ALT-related proteins along with their 30 naturally occurring metabolites. We detected 37 highly significant metabolic and cellular pathways (S8 Table in S1 File; Fig 2). Three metabolic pathways have been primarily related with the ALT mechanism: 1) purine metabolism, 2) cysteine and methionine metabolism, and 3) nicotinate and nicotinamide metabolism (S8 Table in S1 File; Fig 2).

### Networking analysis of ALT-related proteins, pan-cancer oncogenes, and metabolites

**Fig 3:**
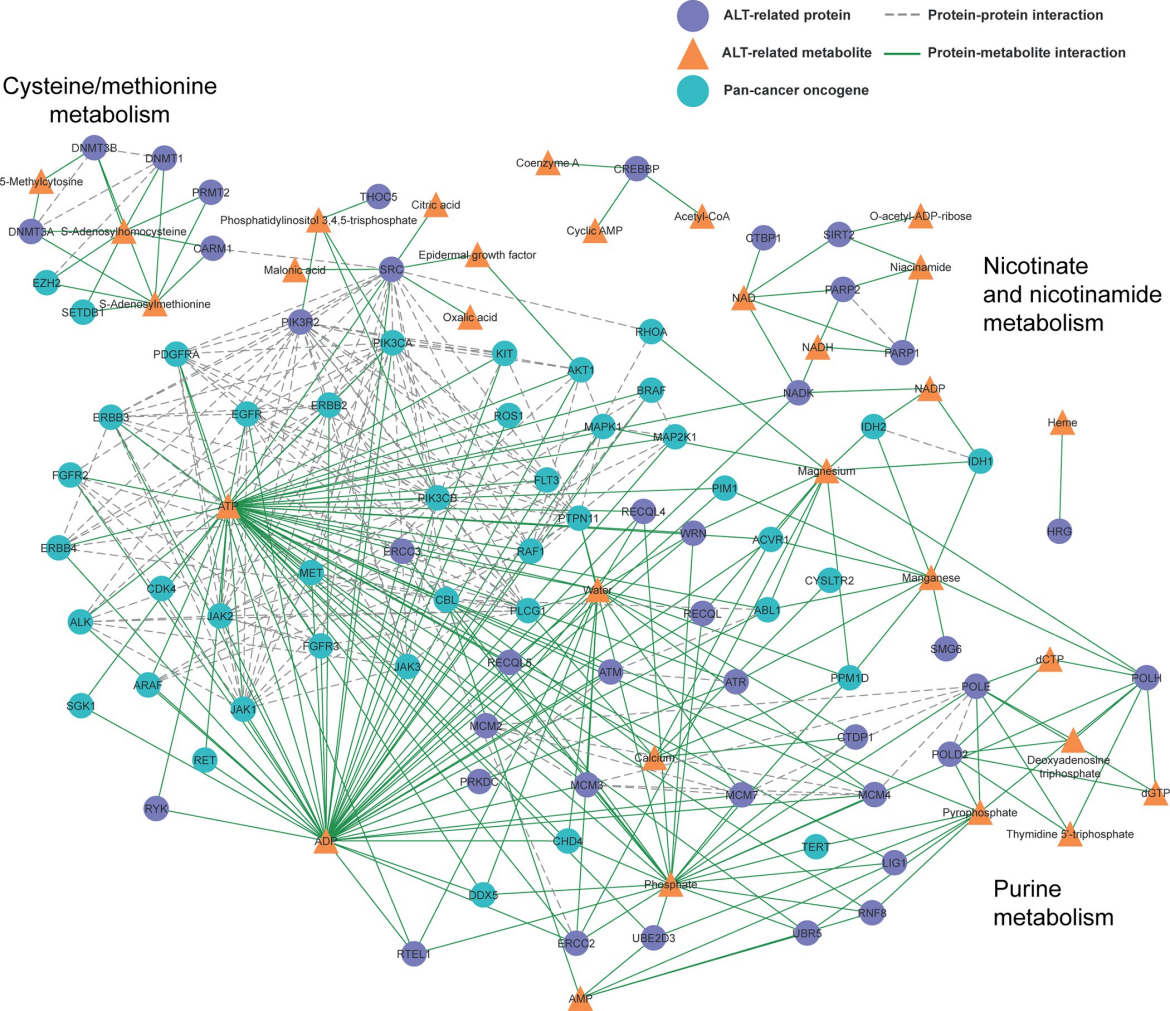
Interaction network of ALT-related proteins, pan-cancer oncogenes, and metabolites. This visual representation depicts the intricate interplay between ALT-related proteins (violet circular nodes), pan-cancer oncogenes (blue circular nodes), and their associated metabolites (orange triangular nodes) across metabolic pathways. The dotted lines indicate protein-protein interactions (PPIs), while solid green lines represent interactions between proteins and metabolites (PMIs). Notably, the pathways of cysteine/methionine metabolism, nicotinate and nicotinamide metabolism, and purine metabolism are highlighted, with specific proteins and metabolites acting as central nodes. This map provides a comprehensive overview of the potential interactions and influential roles these elements may play in the context of ALT and cancer metabolism.

Networking analysis has proved useful in identifying key elements among complex interactions. On this basis, to identify ALT-related metabolic pathways enhanced by pan cancer oncogenes, we next predict protein-metabolite (PMI) and protein-protein interactions (PPIs) between ALT-related proteins, pan-cancer oncogenes, and their corresponding metabolites (Fig 3 and S9 Table in S1 File). The interactions shown in Fig 3 are supported by experimental evidence from various cell lines [22, 28, 29], but remain predictive and require further validation in ALT-specific experimental models.

In our detailed network analysis (Fig 3 and S9 Table in S1 File), we detected intricate interactions between ALT-related proteins, pan-cancer oncogenes, and associated metabolites. Three metabolic pathways emerged as principal components of the network: nicotinate and nicotinamide metabolism, cysteine/methionine metabolism, and purine metabolism. n the Nicotinate and nicotinamide metabolism pathway, key metabolites like Niacinamide, NAD, and NADH revealed pronounced interactions with ALT-related proteins, including pivotal roles for proteins such as SIRT2, PARP1, and PARP2. The Cysteine/methionine metabolism pathway, prominently featured by S-Adenosylmethionine and S-Adenosylhomocysteine metabolites, could be influenced by pan-cancer oncogenes EZH2 and SETDB1. The Purine metabolism pathway, vital for DNA replication and repair [30], showcased a dense interaction network. Metabolites related with this pathway (Table 1) interacts extensively with ALT-related proteins and pan-cancer oncogenes.

### Targeting ALT-related metabolic pathways

To finally detect druggable metabolic pathways aiming to develop treatment strategies for ALT-positive tumors, we next performed an enrichment analysis in MetaboAnalyst 5.0 [23] of the 30 naturally occurring ALT-related metabolites using 461 metabolite sets based on drug pathways from SMPDB [24]. The platform did not find pathway records for three metabolites: HMDB0251846, HMDB0059656 and HMDB0256910. We have found 191 enriched pathways with p-values ≤ 0.0001 (S10 Table in S1 File).

**Fig 4:**
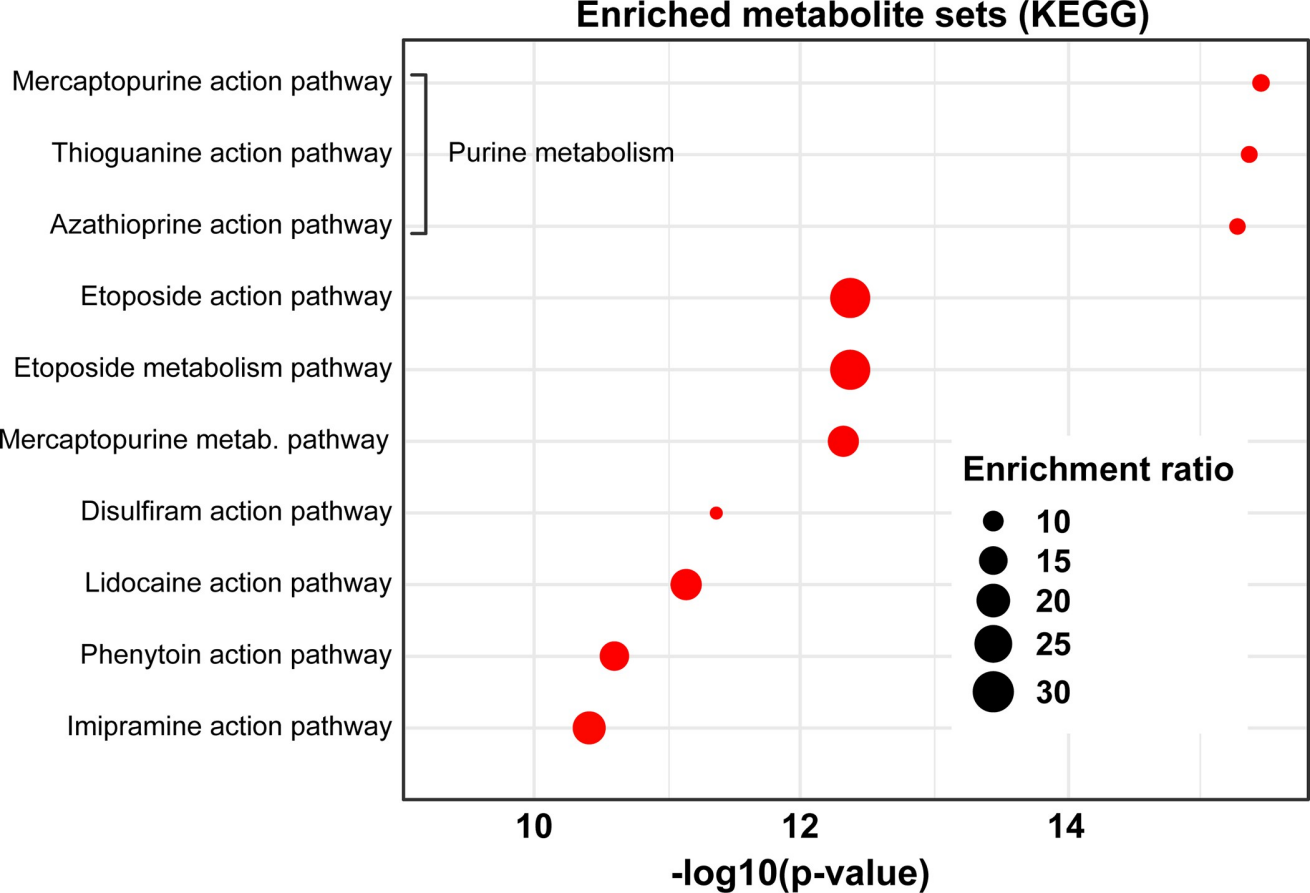
Enrichment of metabolite sets associated with drug action pathways. Scatter plot depicting the enrichment of metabolite sets associated with various metabolic and drug action pathways, as per the KEGG database. The x-axis represents the statistical significance of each set, expressed as -log10(p-value), with a position further to the right indicating increased significance. The size of the data points reflects the enrichment ratio of each set: larger points indicate a higher enrichment ratio. Pathways with high enrichment ratios and low p-values, such as the mercaptopurine action pathway, stand out as potential therapeutic targets of interest.

Fig 4 illustrates the ten most promising druggable metabolic pathways, with purine metabolism prominently positioned as a prime target for possible therapeutic intervention. This central role highlights the potential for utilizing specific agents, such as mercaptopurine, thioguanine, and azathioprine.

Azathioprine, thioguanine, and mercaptopurine are purine antimetabolite prodrugs exerting cytotoxic effects by incorporating their metabolites into DNA and RNA, inhibiting purine nucleotide synthesis, and targeting Ras-related C3 botulinum toxin substrate 1, leading to T cell apoptosis. All undergo metabolic conversions in cells, impacting purine pathways and cellular processes. The shared mechanism involves the inhibition of cellular functions and DNA/RNA incorporation, affecting cell survival and function [31, 32].

## Discussion

In prior studies, we have identified key genes associated with the Alternative Lengthening of Telomeres (ALT) using multi-omic data integration strategies. These genes may be useful as targets for diagnosing and treating ALT-positive cancers [19, 20]. The objective of this study was to identify targetable metabolic pathways for ALT-positive tumors by integrating metabolomics data with our previous findings.

Thus, we first identified 39 ALT-related proteins interacting with 42 distinct metabolites. These metabolites were significantly enriched in five metabolic pathways: 1) nicotinate and nicotinamide metabolism, 2) purine metabolism, 3) citrate cycle (TCA cycle), 4) cysteine and methionine metabolism, and 5) glutathione metabolism (Fig 1). To narrow down the identification of ALT-metabolic pathways, we performed a joint pathway analysis including both genes and metabolites and prioritized three metabolic pathways: 1) nicotinate and nicotinamide metabolism, 2) purine metabolism, and 3) cysteine and methionine metabolism (Fig 2). Noteworthy, we also found pan-cancer oncoproteins that could influence these ALT metabolic pathways (Fig 3). Finally, we identified purine metabolism as a prime target against ALT-positive tumors by using specific agents such as mercaptopurine, thioguanine, and azathioprine (Fig 4).

The comparison of metabolic pathways in mesenchymal-origin tumors vs. those across pan-cancer highlights both shared and unique features of the ALT mechanism. While key pathways such as purine metabolism, nicotinate and nicotinamide metabolism, and citrate cycle metabolism emerged prominently in both mesenchymal and broader tumor contexts, certain metabolic dependencies may be more pronounced in mesenchymal-origin tumors due to their specific cellular background. This alignment suggests that while some metabolic routes are universally associated with ALT-positive tumors, the mesenchymal context may amplify or modulate these pathways, potentially impacting tumor behavior and response to targeted therapies.

PPIs and PMIs presented in Fig 3 were detected experimentally and validated using multiple sources (e.g. PubMed and KEGG). These interactions are curated by experts who review experimental findings and ensure the reliability of the data. Most of these interactions are well-documented and have been identified in cancer cell lines [22, 28, 29]. Specifically, interactions between PARP1, PARP2, and niacinamide are well-known in the synthesis and regulation of NAD+, a crucial coenzyme involved in DNA repair and cellular metabolism. PARP1 and PARP2 use NAD+ as a substrate to carry out poly-ADP-ribosylation, a process critical for DNA damage repair and genomic stability. Niacinamide plays a key role in the NAD+ salvage pathway, where it helps regenerate NAD+ from nicotinamide, thus fueling the activities of PARP enzymes during cellular stress and DNA repair responses [33–36]. Regarding purine metabolism, it is well-stablished that deoxyadenosine triphosphate (dATP), thymidine triphosphate (dTTP), deoxyguanosine triphosphate (dGTP) and deoxycytidine triphosphate (dCTP) are substrates for DNA polymerases POLH, POLE, and POLD2. These components are essential for synthesizing new DNA strands during replication and repair processes in cancer cells [37, 38].

However, it is important to note that these interactions are predictive in nature and still require experimental validation to confirm their occurrence in ALT-specific models. Mass spectrometry-based protein-protein interaction networks could help address this need. Thus, techniques such as affinity purification mass spectrometry (MS), proximity labeling, and cross-linking MS could be used to study PPIs globally [39]. On the other hand, PMIs could also be detected through chemical and structural proteomics approaches or by thermal proteome profiling (TPP) [40].

Purine metabolism is regulated by purinosomes, which are multi-enzyme complexes—composed of nine enzymes—that promotes cancer progression through uncontrolled cell proliferation and increased tumor viability [41–44]. In glioblastoma, alterations in purine metabolism have been associated not only to genomic instability and cell death but also to uncontrolled cell growth [45]. Within purine metabolism, the metabolites dCTP and dGTP are associated with cancer progression. For instance, the dCTPase has been observed to be highly expressed in multiple carcinomas [46], while several studies have demonstrated that dGTP and its derivatives are involved in various aspects of cancer, such as cancer cell survival under oxidative stress, and as targets in chemotherapy and apoptosis induction [47, 48]. Interestingly, we found that dCTP and dGTP interact with ALT-related proteins POLE, POLH, and POLD2, which in turn interact with various ALT proteins. (Fig 3). In addition, several studies have shown that mutations in these proteins promote tumor progression in several cancer types [49, 50].

In glioma, inhibition of purine synthesis has been shown to impaired the self-renewal of brain tumor-initiating cells [51]. This highlights the importance of purine metabolism in cancer treatment and suggest that manipulating this pathway or purinosomes may offer new ways to impair ALT mechanism [52]. In this context, mercaptopurine, thioguanine, and azathioprine could be used to inhibit purine metabolism and induce cancer cell death (Fig 4).

Regarding the nicotinate and nicotinamide metabolism pathway, the nicotinamide adenine dinucleotide (NAD+) coenzyme regulates various cellular processes such as cellular energy production, DNA repair, telomerase activity, cell growth, and cell death, thereby linking it to tumor growth and survival [53, 54]; its deregulation, a common phenomenon in diverse types of cancer, can trigger several events that favor tumor development and progression [53]. This is exemplified in glioblastoma, where the overexpression of nicotinamide N-methyltransferase (NNMT) depletes the methyl donor S-adenosylmethionine (SAM), thereby promoting tumor growth [55]. It has also been shown that the expression of nicotinamide phosphoribosyltransferase (NAMPT), an essential enzyme for NAD+ synthesis, is increased in the most aggressive and invasive cancers and in tumor metastases [53]. Additionally, NAD+ participates in the base excision repair (BER) pathway, which operates through the PARP-NAD-SIRT axis [56]. This aligns with our results presented in Fig 3, where NAD+ interacts with ALT-related proteins: PARP1, PARP2, and SIRT2. Thus, targeting NAD+ metabolism could induce cytotoxicity in cancer cells [54].

Concerning cysteine and methionine metabolism, it has been observed that alterations in this pathway contributes to tumorigenesis by facilitating epigenetic regulation of gene expression [57]. Furthermore, this has also been observed in different cancer cell lines, suggesting a link with oncogenesis [57]. Moreover, some cancer types are auxotrophic for methionine due to alterations in methionine metabolism, which has been used for therapeutic approaches [58].

In our analysis related with this pathway (Fig 3), ALT-related proteins DNMT1, DNMT3A, DNMT3B, CARM1, and PRMT2 interact with S-Adenosylmethionine and S-Adenosylhomocysteine metabolites. Noteworthy, several studies have shown that alterations in these proteins contribute to tumorigenesis [59–65]. For instance, the CARM1 protein plays a multifaceted role in cancer metabolism, affecting metabolic pathways, regulating gene expression, and promoting metastasis [62, 63]. Conversely, the PRMT2 protein is implicated in cancer metabolism through the modulation of hormonal receptor signaling, gene expression, and cell cycle regulation [64, 65]. Moreover, this pathway could be influenced by two CDGs, EZH2 and SETDB1. Indeed, SETDB1, a histone methyltransferase, promotes heterochromatin formation at telomeres. This, in turn, stimulates transcriptional elongation at telomeres and the recruitment of essential ALT factors [66]. Moreover, targeting EZH2 for cancer therapy is currently a highly active area of research, and various types of EZH2 inhibitors have been developed [67].

This study indirectly prioritized three metabolic pathways related to the ALT process and identified potential therapeutic targets. These findings point to the need for more research into these metabolic pathways and how they affect the growth of ALT-positive tumors. Thus, this research could lead to the development of new therapeutic approaches to combat this tumoral process. While this work provides a wider understanding of the metabolomic aspects of ALT-positive cancers, further experimental research is needed to elucidate the functional roles of these metabolites in this process.

## Supporting information

S1 File A single file containing S1 to S10 Tables. Each table is numbered and labeled within the document.
(XLSX)

S1 Fig Identification of ALT proteins engaged in protein-metabolite interactions. A workflow detailing the identification, filtration, and prioritization of ALT-related genes and their interactions with metabolites in pan-cancer and tumors from mesenchymal origin. ALT = Alternative Lengthening of Telomeres; APB = ALT-associated PML Bodies; PMIs = Protein-Metabolite interactions.
(PDF)

S2 Fig ALT-related metabolic pathways in soft tissue tumors. Enrichment analysis of ALT-related metabolites using KEGG (A) and SMPDB (B) databases. Metabolite pathways are listed on the y-axis with their respective significance levels shown on the x-axis, represented as the negative logarithm (base 10) of the p-value. The size of the circles is proportional to the enrichment ratio, with larger circles indicating higher enrichment. (C) Venn diagram displaying shared statistically significant pathways between SMPDB and KEGG.
(PDF)
