# Supplementary figures and images for: Metabolic pathways of Alternative Lengthening of Telomeres in pan-carcinoma
Source: PLoS One. 2025 Feb 21;20(2):e0314012. doi: 10.1371/journal.pone.0314012 (PMC11845024; doi:10.1371/journal.pone.0314012)

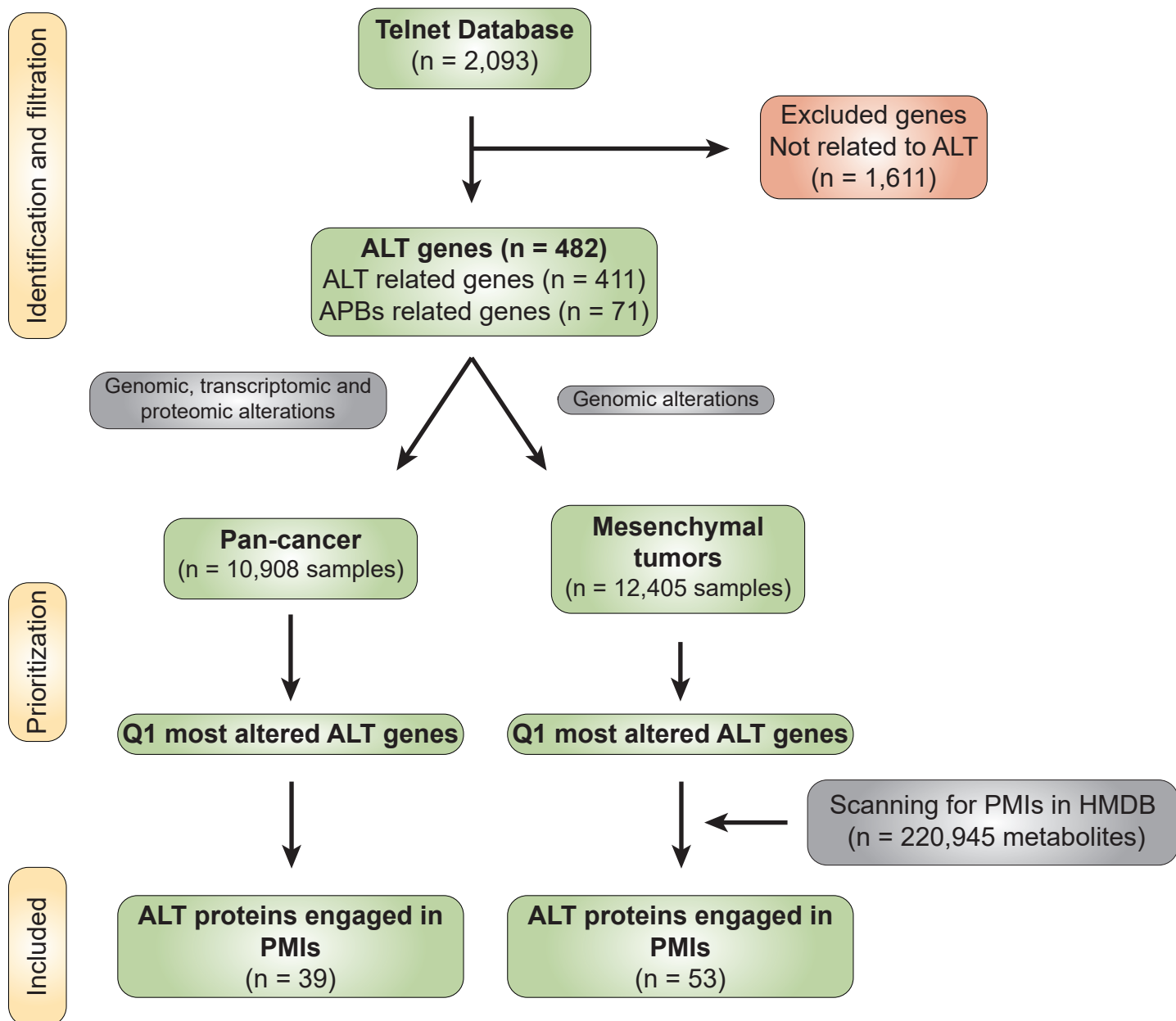

Supplement: S1 Fig — A workflow detailing the identification, filtration, and prioritization of ALT-related genes and their interactions with metabolites in pan-cancer and tumors from mesenchymal origin. ALT = Alternative Lengthening of Telomeres; APB = ALT-associated PML Bodies; PMIs = Protein-Metabolite interactions. (PDF) [file pone.0314012.s002.pdf]

A

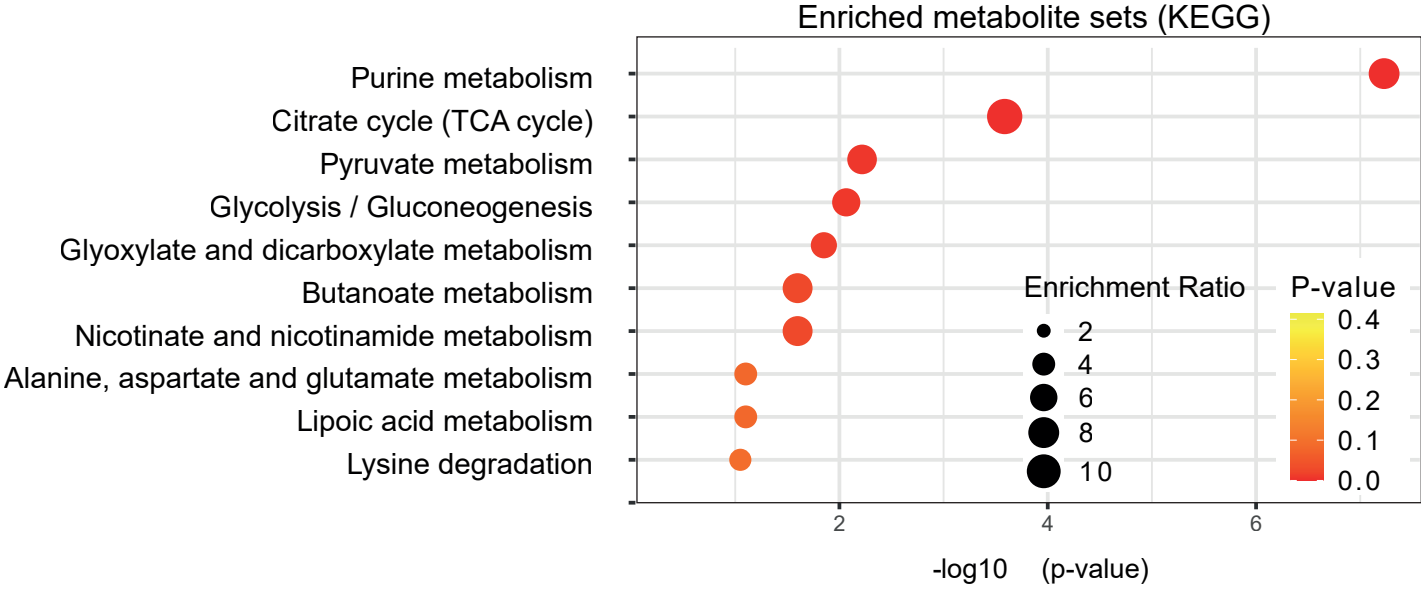

B

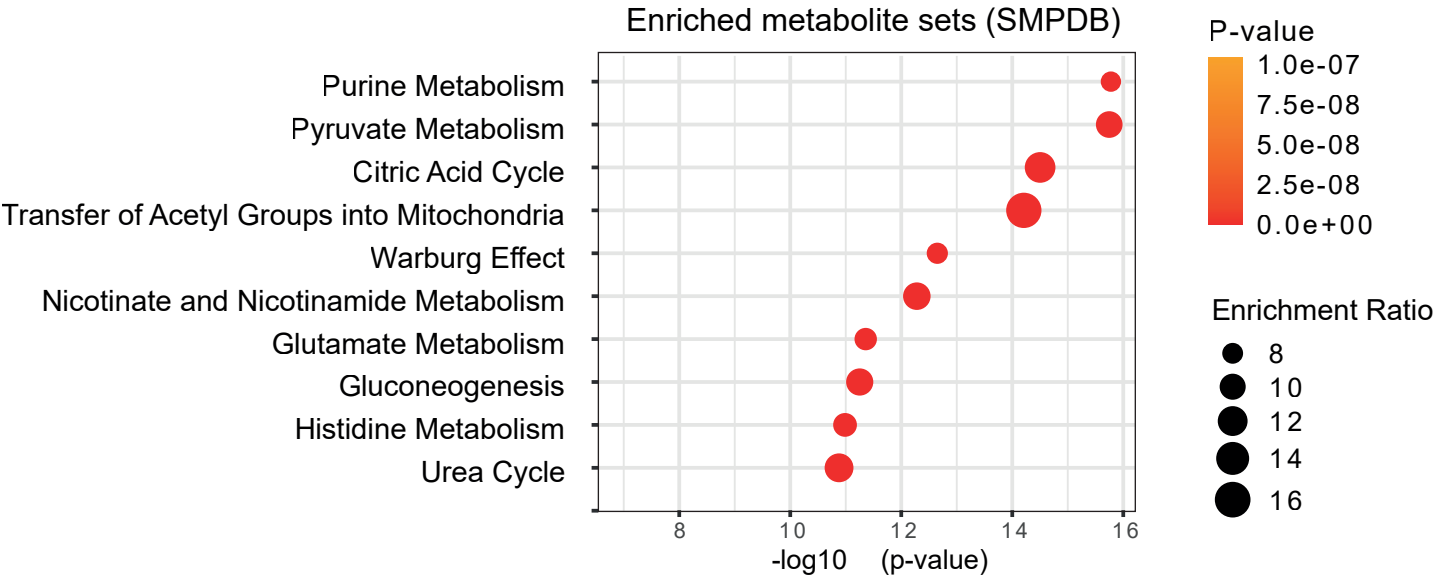

C

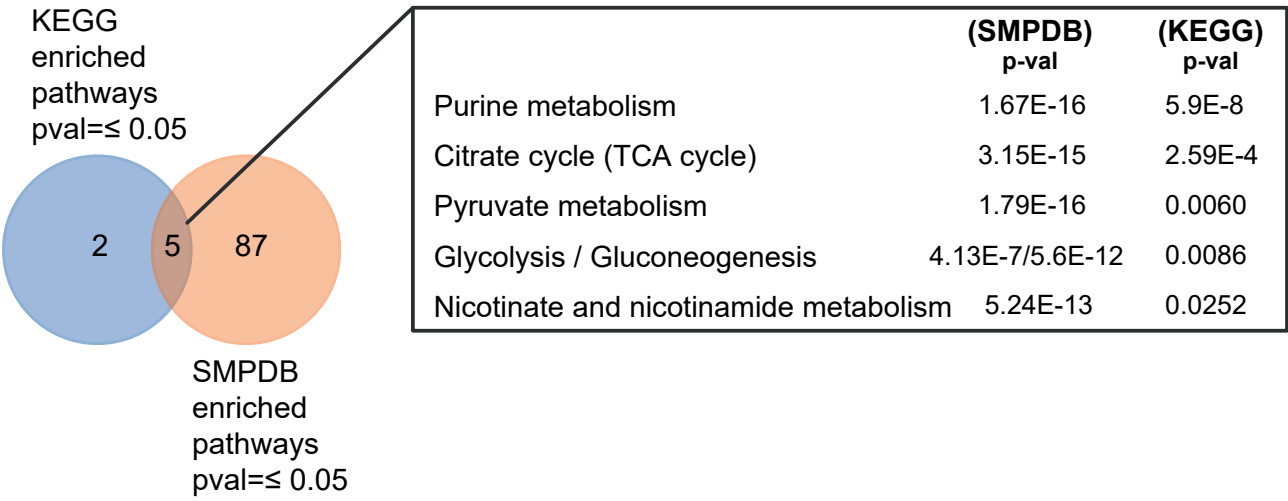

Supplement: S2 Fig — Enrichment analysis of ALT-related metabolites using KEGG (A) and SMPDB (B) databases. Metabolite pathways are listed on the y-axis with their respective significance levels shown on the x-axis, represented as the negative logarithm (base 10) of the p-value. The size of the circles is proportional to the enrichment ratio, with larger circles indicating higher enrichment. (C) Venn diagram displaying shared statistically significant pathways between SMPDB and KEGG. (PDF) [file pone.0314012.s003.pdf]
